# Comparison of Different Test Systems for the Detection of Antiphospholipid Antibodies in a Chinese Cohort

**DOI:** 10.3389/fimmu.2021.648881

**Published:** 2021-07-02

**Authors:** Chaojun Hu, Siting Li, Zhijuan Xie, Hanxiao You, Hui Jiang, Yu Shi, Wanting Qi, Jiuliang Zhao, Qian Wang, Xinping Tian, Mengtao Li, Yan Zhao, Xiaofeng Zeng

**Affiliations:** ^1^ Department of Rheumatology, Peking Union Medical College Hospital, Peking Union Medical College & Chinese Academy of Medical Sciences, Key Laboratory of Rheumatology & Clinical Immunology, Ministry of Education, Beijing, China; ^2^ National Clinical Research Center for Dermatologic and Immunologic Diseases (NCRC-DID), Beijing, China

**Keywords:** antiphospholipid antibodies, antiphospholipid syndrome, chemiluminescent immunoassay, enzyme-linked immunosorbent assay, anti-*β*2 glycoprotein-I, anti-cardiolipin

## Abstract

**Background:**

Diagnosis of antiphospholipid syndrome (APS) is based on the positivity of laboratory criteria antiphospholipid antibodies (aPLs). Test results for aPLs could be contradictory among different detection methods as well as commercial manufacturers. This study aimed to assess and compare the diagnostic and analytic performances of four commercial assays prevalently used in China.

**Methods:**

A total of 313 patients including 100 patients diagnosed with primary APS, 52 with APS secondary to SLE, 71 with SLE, and 90 health controls were recruited. Serum IgG, IgM, and IgA for aCL, and a*β*2GPI antibodies were detected with two ELISA and two CLIA systems, and test system with the best diagnostic value was explored of its correlation with key clinical features.

**Results:**

CLIA by YHLO Biotech Co. was considered as the system with the best predictive power, where 58.55 and 57.89% of APS patients were positive for aCL or a*β*2GPI for at least one antibody (IgG or IgM or IgA). Overall, CLIA showed better performance characteristics than traditional ELISA test systems.

**Conclusion:**

CLIA was considered as a better platform for aPL detection in APS diagnosis. A combination of other detection platforms could assist in differential diagnosis as well as in identifying high-risk patients.

## Introduction

The antiphospholipid syndrome (APS) is defined by the development of venous/arterial thromboses or by the occurrence of obstetrical events including recurrent fetal losses or increased perinatal morbidity, with the persistent presence of antiphospholipid antibodies (aPLs). According to the 2006 APS classification criteria, APS diagnosis is based on the positivity of at least one of the clinical criteria as well as one of laboratory criteria including lupus anticoagulant (LA), high level of anti-cardiolipin (aCL), anti-*β*2 glycoprotein-I (a*β*2GPI) immunoglobulin isotype G (IgG) or M (IgM) (1). More recently, non-criteria aPLs including anti-aCL or anti-b2GPI IgA, anti-phosphatidylserine–prothrombin (aPS/PT) complex, anti-annexin A5 antibodies (aAnxV), *etc.* are receiving increasing attention (2).

APS could be associated with several severe clinical outcomes such as pulmonary embolism, acute myocardial infarction, and stroke, which demand immediate appropriate intervention. On the other hand, anticoagulant treatment commonly utilized for APS could increase bleeding risk for susceptible patients. Since aPL detection comprise a large part of APS diagnosis, a detection system with high sensitivity and specificity is required in order to timely identify APS patients as well as provide accurate clinical intervention (3). Besides, evaluation of aPLs could also contribute to prognosis and risk assessment for associated clinical manifestations (4, 5).

Numerous guidelines and studies concerning aCL and a*β*2GPI tests have been published (6). However, test results for aPLs remain contradictory among different detection methods as well as commercial manufacturers, probably due to the lack of standardization for cut-off values, method of calibration and quantitation, choice of solid phase and coating, type and source of antigen, and other analytic problems (7–9). Traditionally, enzyme-linked immunosorbent assay (ELISA) was applied due to its relative time and cost-efficiency. In recent years, novel automating detection systems, such as chemiluminescent immunoassay (CLIA), addressable laser bead immunoassay (ALBIA), line immunoassay (LIA), *etc.* have been introduced for aPL detection, and promising results have been yielded (10–14). Automatization can improve the reproducibility and reduce interlaboratory variation, yet may show distinct performance characteristics compared to ELISA (15, 16).

More specifically, in China, home-conducted ELISA is still most widely applied at laboratories for APS diagnosis. However, an increasing number of automated analyzers have been equipped by large general hospitals with high application potentials. Regarding commercially available systems, most studies focused on measuring and comparing only one assay to laboratory-conducted ELISA (17). However, little attention has been paid to simultaneously evaluate different test systems that are commonly chosen. The aim of this study was to assess and compare the diagnostic and analytic performances of four commercial assays prevalently used in China, including two ELISA and two CLIA systems, in a Chinese prospective APS cohort. Detection of IgG, IgM, and IgA for aCL and a*β*2GPI antibodies was evaluated, and a test system with the best diagnostic value was explored of its correlation with key clinical features.

## Patients and Methods

### Patients Recruitment

This was a single-center, prospective cohort study conducted at Peking Union Medical College Hospital (PUMCH) and the National Clinical Research Center for Dermatologic and Immunologic Diseases (NCRC-DID) from May 2017 to January 2020. A total of 313 consecutive patients were included in this study, of which 100 patients had been diagnosed with primary APS (PAPS group), 52 with APS secondary to SLE (SAPS group), 71 with SLE (SLE group), and 90 healthy controls (HC group). Diagnosis of APS was defined by clinicians according to the 2006 Sydney revised classification criteria (1). According to the criteria, IgG and IgM aCL and a2GPI were analyzed with standardized ELISA (INOVA Diagnostics) at the Key Laboratory. Lupus anticoagulant was detected and evaluated according to the ISTH recommendations. Dilute Russell viper venom time (dRVVT) testing and activated partial thromboplastin time were measured, where LAC was considered positive if the ratio of screen/confirm time ratio was >1.20. Diagnosis of SLE was based on the 1997 ACR criteria and confirmed by the 2019 EULAR/ACR criteria. Clinical manifestations were recorded for PAPS, SAPS, and SLE groups, including vascular thrombosis (arterial or venous), pregnancy morbidity, and extra-criteria manifestations, including thrombocytopenia, heart valve disease, autoimmune hemolytic anemia, and neurological disorders, *etc.* For the HC group, only aPL serology information was present. For each subject, 4 ml of blood was collected with the help of a BD vacutainer without anticoagulants. Blood samples were allowed to clot at room temperature for 1 h and then centrifuged at 4°C for 5 min at 3,000 rpm. Serum was collected and stored at −80°C. No sample was exposed to more than one freeze–thaw cycle before analysis. The study was approved by the ethics committee at PUMCH and fulfilled the ethical guidelines of the declaration of Helsinki. All subjects gave written informed consent.

### Laboratory Tests

For each study subject, IgG, IgM, and IgA isotypes of aCL and a*β*2GPI were analyzed with four systems listed below: a. iFlash CLIA kits provided by YHLO Biotech Co., Shenzhen, China (Y-CLIA); b. QUANTA Flash^®^ CLIA kits provided by INOVA Diagnostics, Inc., San Diego, CA, US, Werfen Group as sales agent (W-CLIA); c. QUANTA Lite™ ELISA kits provided by INOVA Diagnostics, Inc., San Diego, CA, US, Werfen Group as sales agent (W-ELISA); d. AESKULISA^®^ ELISA test kits provided by Aesku.Diagnostics GmbH & Co. KG, Wendelsheim, Germany (A-ELISA). Detailed characteristics of test systems from different manufacturers were summarized in **Table 2**. Cut-off values were defined for each system as recommended by the manufacturer.

### Statistical Analysis

Statistical analysis was performed using SPSS 26.0 or R (version 3.6.2). The χ^2^ test or Fisher’s exact test was used for comparison of categorical variables, and Wilcoxon test was used for continuous variables after normality was explored with the Shapiro–Wilk test. Sensitivities, specificities, and accuracies in APS diagnosis were compared in the McNemar test. Youden Index, positive and negative predictive values (PPV and NPV), and odds ratio (OR) with 95% confidence interval (95% CI) were also shown. Correlation of different aPL isotype levels with clinical manifestations was calculated, and clinical events with 95% CI were displayed. Two-tailed values of p less than 0.05 were considered statistically significant.

## Results

### Patient Characteristics

Among 152 APS patients, there were 63 (63.0%) females for PAPS, 46 (88.5%) for SAPS, and the mean age for each was 36.3 and 32.9 years (**Table 1**). Mean age was 30.1+/−8.2 years in the SLE group, of which 61 (85.9%) were female, while the HC group had 41 (45.6%) female and a mean age of 43.4+/−12.2. Detailed clinical manifestations were recorded for both APS and SLE patients and were shown. Thrombosis was most commonly present, with 80 (80.0%) for PAPS and 39 (75%) for SAPS, but not in the SLE group. Patients with history of arterial or venous thrombosis were recorded for APS patients. Pregnancy morbidity, history of adverse pregnancy, microangiopathy, and LA were also observed in both PAPS and SAPS group. Of all the clinical manifestations, the prevalence of thrombocytopenia was significantly different between PAPS and SAPS group (χ^2^ = 4.382, p = 0.036).

**Table 1:** Demographic and clinical variables of subjects (n = 313).

	APS (152)		SLE (71)	Health controls (90)
	Primary (100)	Secondary (52)		
Gender (female/male)	63/37	46/6	61/10	41/49
Mean age (years ± SD)	36.3 ± 12.1	32.9 ± 10.2	30.1 ± 8.2	43.4 ± 12.2
**Clinical manifestations**				
Thrombosis, n (%)	80 (80.0%)	39 (75.0%)	0	NA
Pregnancy morbidity, n (%)	33 (33.0%)	17 (32.7%)	0	NA
Thrombosis + pregnancy morbidity, n (%)	13 (13.0%)	4 (7.7%)	0	NA
LA, n (%)	73 (73.0%)	44 (84.6%)	17 (23.9%)	NA
History of arterial thrombosis, n (%)	43 (43.0%)	21 (40.4%)	0	NA
Stroke, n (%)	4 (4.0%)	2 (3.8%)	0	NA
Coronary heart disease, n (%)	9 (9.0%)	2 (3.8%)	0	NA
Eye involvement, n (%)	3 (3.0%)	1 (1.9%)		
Lower limb artery occlusion, n (%)	1 (1.0%)	0	0	NA
History of venous thrombosis, n (%)	47 (47.0%)	24 (46.2%)	0	NA
Deep vein thrombosis, n (%)	19 (19.0%)	7 (13.5%)	0	NA
Pulmonary embolism, n (%)	19 (19.0%)	2 (3.8%)	0	NA
Upper limb vein thrombosis, n (%)	0	1 (1.9%)	0	NA
Renal vein thrombosis, n (%)	1 (1.0%)	0	0	NA
Portal vein thrombosis, n (%)	4 (4.0%)	1 (1.9%)	0	NA
Cerebral venous and sinus thrombosis, n (%)	3 (3.0%)	1 (1.9%)	0	NA
Central retinal venous occlusion, n (%)	1 (1.0%)	0	0	NA
Microangiopathy, n (%)	57 (57.0%)	24 (46.2%)	0	NA
Thrombocytopenia, n (%)	38 (38.0%)	*29 (55.8%)	21 (29.6%)	NA
Heat valve disease, n (%)	0	6 (11.5%)	0	NA
Non-stroke CNS manifestations, n (%)	4 (4.0%)	4 (7.7%)	0	NA
Antiphospholipid syndrome nephropathy, n (%)	6 (6.0%)	2 (3.8%)	0	NA
Autoimmune hemolytic anemia, n (%)	1 (1.0%)	5 (9.6%)	0	NA
Thrombotic Microangiopathy, n (%)	0	1 (1.9%)	0	NA
Hemolytic uremic syndrome, n (%)	1 (1.0%)	0	0	NA
History of adverse pregnancy, n (%)	37 (37.0%)	21 (40.4%)	4 (5.6%)	NA
Early fetal loss (<10 weeks), n (%)	12 (12.0%)	8 (15.4%)	4 (5.6%)	NA
Late fetal loss (10–28 weeks), n (%)	19 (19.0%)	12 (23.1%)	0	NA
Placenta insufficiency, n (%)	14 (14.0%)	7 (13.5%)	0	NA

*p = 0.036, significant different from primary APS. NA, Not Available.

### Assay Characteristics

As summarized in **Table 2**, the coating, conjugation, calibration, and cut-off values with their calculation were listed for four commercial test systems. More specifically, Y-CLIA conducted paramagnetic particle chemiluminescent immunoassay using a fully automated iFlash 3000 Chemiluminescence Immunoassay Analyzer. Recommended values with best sensitivity, specificity, and false positive results of healthy donors against APS, SLE, and other autoimmune disease patients were chosen for all antibody isotypes. For W-CLIA, antigen-specific paramagnetic bead chemiluminescent immunoassay was conducted employing the fully automated BIO-FLASH CLIA instrument. Cut-off values for all antibodies were calculated using the 99th percentile in healthy groups. W-ELISA was a semi-quantitative enzyme linked immunosorbent assay manually conducted according to the manufacturer’s instruction.

Cut-off values were set based on the evaluation of normal and positive antibody samples. For A-ELISA, assay was also manually conducted following manufacturer’s protocols, yet no information was provided for cut-off value calculation.

**Table 2:** Characteristics of test systems from different manufacturers.

	Assay	Coating well/particle	Conjugate	Calibration	Manufacturer’s cutoff	Calculation
iFlash (Y-CLIA)	Anti-cardiolipin IgM, IgG, IgA	Human cardiolipin	Acridine anti-human IgM/IgG/IgA antibody	Internal standard: Louisville APL Diagnostics	10 U/ml	59/38 APS and other AID patients, 241/262 blood bank donors, a recommended value
	Anti-*β*2 glycoprotein I IgM, IgG, IgA	Human *β*2 glycoprotein I	Acridine anti-human IgM/IgG/IgA antibody	Internal standard: Louisville APL Diagnostics	20 U/ml	62/72 APS and other AID patients, 238/308 blood bank donors, a recommended value
QUANTA Flash (W-CLIA)	QUANTA Flash aCL IgG, IgM, IgA	Bovine cardiolipin with human *β*2GPI	Isoluminol anti-human IgM/IgG/IgA antibody	Internal standard: HCAL for IgG and EY2C9 for IgM	20 CU	250/262 blood bank donors, 99th percentile
	QUANTA Flash *β*2GP1 IgG, IgM, IgA	Human *β*2GPI	Isoluminol anti-human IgM/IgG/IgA antibody	Internal standard: HCAL for IgG and IgA, EY2C9 for IgM	20 CU	250–252 blood bank donors, 99th percentile
QUANTA Lite (W-ELISA)	QUANTA Lite ACA IgG III, IgM III, IgAIII	Purified cardiolipin and bovine *β*2GPI	HRP goat anti-human IgM/IgG/IgA antibody	Internal standard: HCAL for IgG and EY2C9 for IgM	20 MPL, GPL, APL	488–489 normal donors, a recommended value
	QUANTA Lite *β*2 GPI IgG, IgM, IgA	Purified *β*2GPI	HRP goat anti-human IgM/IgG/IgA antibody	Internal standard: human serum antibodies to *β*2GPI	20 SMU, SGU, SAU	11–313 normal donors, a recommended value
AESKULISA (A-ELISA)	AESKULISA Cardiolipin-GM, Cardiolipin-A	Purified cardiolipin and bovine *β*2GPI	HRP anti-human IgM/IgG/IgA antibody	Internal standard: HCAL for IgG and EY2C9 for IgM, Louisville APL for IgA	18 MPL, GPL, APL	NA
	AESKULISA *β*2-Glyco-GM, *β*2-Glyco-A	Purified *β*2GPI	HRP anti-human IgM/IgG/IgA antibody	Internal standard: HCAL for IgG and EY2C9 for IgM	18 U/ml	NA

MPL, GPL, and APL for IgM, IgG, and IgA phospholipid units; SMU, SGU, SAU for standard IgM, IgG, and IgA units, HRP for horseradish peroxidase. NA, Not Available.

### Predictive Power of aPLs for Different Test Systems

Antibody results obtained from four test systems were evaluated for diagnostic power with sensitivity, specificity, accuracy, Youden Index, PPV, and NPV in APS diagnosis from the HC group in **Table 3**. For each antibody type, sensitivity, specificity, and accuracy were compared first between the same test methods (*i.e.*, Y-CLIA against W-CLIA, W-ELISA against A-ELISA). The better system from each method, if identified, was then compared to determine the best system, which was further evaluated for clinical manifestation prediction. As shown in **Table 3**, the accuracy of aCL IgG was significantly higher for Y-CLIA than W-CLIA (p < 0.001), and A-ELISA than W-ELISA (p = 0.035). The sensitivity (p < 0.001) and accuracy (p < 0.001) were both significantly higher for Y-CLIA method. For aCL IgM, sensitivity and accuracy were significantly higher for W-ELISA than A-ELISA (p < 0.001). As for aCL IgA, Y-CLIA and A-ELISA were selected respectively for comparison, and the specificity of the former was significantly higher (p = 0.031). Sensitivity and accuracy of positivity of aCL IgG, IgM, or IgA were also significantly higher for Y-CLIA than for W-CLIA (p < 0.001). Y-CLIA and W-ELISA were selected as better systems for positivity of aCL IgG or IgM, and significant difference was observed for accuracy (p = 0.022). Concerning a*β*2GPI, Y-CLIA and W-CLIA were selected for comparison of IgM, whose specificity (p = 0.049) was higher that the former. Sensitivity and accuracy of positivity of a*β*2GPI IgG, IgM, or IgA, as well as those of aCL IgG or IgM, were all significantly higher for Y-CLIA. All in all, Y-CLIA was considered as a system with the best predictive power.

**Table 3:** Comparison of the predictive power of aPL tests from different test systems in APS diagnosis.

			Sensitivity (%)	Specificity (%)	Accuracy (%)	Youden Index	PPV (%)	NPV (%)	
aCL IgG	CLIA	#Y*	50.66	100.00	69.01	0.507	100.00		54.55
		W	40.13	95.45	60.42	0.356	93.85		48.00
	p_C_		1.000	0.134	**<0.001**				
	ELISA	W	37.50	95.12	57.69	0.326	93.44		45.09
		A*	37.09	100.00	60.58	0.371	100		48.65
	p_E_		1.000	1.000	**0.035**				
	p_C/E_		**<0.001**	NA	**<0.001**				
aCL IgM	CLIA	Y	16.45	96.67	46.28	0.131	89.29		40.65
		W	13.82	100.00	44.95	0.138	100.00		39.63
	p_C_		0.344	0.250	0.332				
	ELISA	W*	33.55	98.78	56.41	0.324	98.08		44.51
		A	8.61	97.78	41.90	0.064	86.67		38.94
	p_E_		**<0.001**	1.000	**<0.001**				
	p_C/E_								
aCL IgA	CLIA	#Y*	23.03	98.89	51.24	0.219	97.22		43.20
		W	13.82	100.00	44.49	0.138	100.00		39.07
	p_C_		**0.001**	1.000	**<0.001**				
	ELISA	W	4.61	98.78	37.61	0.034	87.50		35.84
		A*	30.46	92.22	53.5	0.227	86.79		44.15
	p_E_		**<0.001**	0.063	**<0.001**				
	p_C/E_		0.071	**0.031**	0.511				
aCL IgG or IgM or IgA	CLIA	Y*	58.55	95.56	72.32	0.542	95.70		57.72
		W	46.71	95.24	64.08	0.419	94.67		49.69
	p_C_		**<0.001**	1.000	**<0.001**				
	ELISA	W	53.95	93.75	67.77	0.477	94.25		51.72
		A	51.66	91.11	66.39	0.428	90.70		52.90
	p_E_		0.608	1.000	0.775				
	p_C/E_								
aCL IgG or IgM	CLIA	#Y*	58.55	96.67	72.73	0.553	96.74		58.00
		W	46.05	95.29	63.72	0.414	94.59		49.69
	p_C_		**<0.001**	0.625	**<0.001**				
	ELISA	W*	53.95	95.00	68.10	0.489	95.35		52.05
		A	41.06	97.78	62.24	0.389	96.88		49.72
	p_E_		**0.001**	0.375	0.302				
	p_C/E_		0.349	0.687	**0.022**				
a*β*2GPI IgG	CLIA	Y	46.71	100.00	66.53	0.467	100.00		52.63
		W	50.00	97.67	67.22	0.477	97.44		52.50
	p_C_		0.442	0.500	1.000				
	ELISA	W*	31.58	95.51	55.19	0.271	92.31		44.97
		A	23.18	100.00	51.86	0.232	100.00		43.69
	p_E_		**0.004**	0.125	0.152				
	p_C/E_								
a*β*2GPI IgM	CLIA	#Y*	21.1	97.78	49.58	0.189	94.12		42.31
		W	9.21	100.00	42.26	0.092	100.00		38.67
	p_C_		**<0.001**	0.500	**<0.001**				
	ELISA	W*	15.13	100.00	46.02	0.151	100.00		40.28
		A	7.95	98.89	41.91	0.068	92.31		39.04
	p_E_		**0.021**	1.000	0.064				
	p_C/E_		**0.049**	NA	0.136				
a*β*2GPI IgA	CLIA	Y	16.45	98.89	47.11	0.153	96.15		41.20
		W	13.82	100.00	44.95	0.138	100.00		39.63
	p_C_		0.344	1.000	0.118				
	ELISA	W*	11.84	96.51	42.43	0.083	85.71		38.25
		A	6.62	98.89	41.08	0.055	90.91		38.70
	p_E_		**0.039**	0.500	0.815				
	p_C/E_								
a*β*2GPI IgG or IgM or IgA	CLIA	#Y*	57.89	96.67	72.31	0.546	96.70		57.62
		W	51.32	97.62	67.80	0.489	97.50		52.56
	p_C_		0.110	1.000	**0.028**				
	ELISA	W*	42.11	95.29	61.18	0.374	94.12		47.93
		A	31.79	97.78	56.43	0.296	96.00		46.07
	p_E_		**0.003**	0.250	0.150				
	p_C/E_		**<0.001**	1.000	**<0.001**				
a*β*2GPI IgG or IgM	CLIA	#Y*	57.24	97.75	72.31	0.55	97.75		57.52
		W	51.32	97.65	67.93	0.489	97.50		52.87
	p_C_		0.163	1.000	**0.038**				
	ELISA	W*	39.47	96.55	60.25	0.364	95.24		47.73
		A	29.14	98.89	55.19	0.28	97.78		45.41
	p_E_		**0.003**	0.250	0.061				
	p_C/E_		**<0.001**	1.000	**<0.001**				

P-values of sensitivity and specificity are calculated with McNemar test; significant results are marked bold.

P_C_, Comparison of CLIA results from different manufacturers. *Better results.

P_E_, Comparison of ELISA results from different manufacturers. *Better results.

P_C/E_, Comparison of better CLIA and ELISA results. #Best results.

Odds ratios (ORs) with 95% confidence intervals (CIs) are shown.

Similarly, the sensitivity, specificity, and accuracy were also compared among four systems in identifying thrombosis and pregnancy morbidity (**Table 4**). For thrombosis events, significant results for sensitivity and accuracy of aCL and a*β*2GPI positivity were all higher for Y-CLIA than for W-CLIA and for W-ELISA than for A-ELISA. Y-CLIA still showed higher accuracy (p = 0.022 for aCL IgG or IgM and p = 0.001 for a*β*2GPI IgM). As for pregnancy morbidity, significant results for specificity and accuracy of aCL and a*β*2GPI positivity were significantly higher for W-CLIA than for Y-CLIA and for A-ELISA than for W-ELISA.

**Table 4:** Comparison of the predictive power of aPL tests from different test systems for criterial manifestations.

			Thrombosis				Pregnancy morbidity		
			Sensitivity (%)	Specificity (%)	Accuracy (%)		Sensitivity (%)	Specificity (%)	Accuracy (%)
aCL IgG	CLIA	Y*	52.94	58.06	54	Y	48	44.07	45.87
		W	43.67	70.97	49.34	W*	38	61.02	50.46
	p_C_		**0.007**	0.125	**0.001**		0.125	**0.006**	**0.001**
	ELISA	W	40.34	71	46.67	W	32	62.71	48.62
		A	38.98	67.74	44.97	A	38.78	66.1	53.7
	p_E_		0.754	1	1		0.25	0.625	1
	p_C/E_								
aCL IgM	CLIA	Y	16.81	83.87	30.66	Y	16	84.75	53.21
		W	14.29	87.1	29.33	W	12	84.75	51.37
	p_C_		1	0.453	0.344		0.687	1	0.727
	ELISA	W	31.09	54.84	36	W	40	67.8	48.62
		A	7.63	87.1	24.16	A	10.2	89.83	53.7
	p_E_		**<0.001**	**0.006**	**<0.001**		**<0.001**	**<0.001**	**<0.001**
	p_C/E_								
aCL IgA	CLIA	Y*	22.69	74.19	33.33	Y	26	76.27	53.21
		W	12.61	80.65	26.67	W*	20	88.14	56.88
	p_C_		**0.004**	0.5	**0.001**		0.375	**0.039**	**0.013**
	ELISA	W	2.52	87.1	20	W	8	96.61	55.96
		A	29.66	64.52	36.91	A	34.69	64.41	50.93
	p_E_		**<0.001**	**0.016**	**<0.001**		**<0.001**	**<0.001**	**<0.001**
	p_C/E_								
aCL IgG or IgM or IgA	CLIA	Y*	62.18	54.84	60.66	Y	52	35.59	43.12
		W	50.42	64.52	53.33	W*	42	52.54	47.71
	p_C_		**0.001**	0.25	**<0.001**		0.125	**0.002**	**<0.001**
	ELISA	W	55.46	48.39	54	W	52	45.76	48.62
		A	53.39	51.61	53.02	A	51.02	49.15	50
	p_E_		0.69	1	0.608		1	0.791	0.701
	p_C/E_								
aCL IgG or IgM	CLIA	#Y*	62.18	54.84	60.66	Y	52	35.59	43.12
		W	50.42	67.74	54	W*	40	52.54	46.79
	p_C_		**0.001**	0.125	**<0.001**		0.07	**0.002**	**<0.001**
	ELISA	W*	55.46	48.39	54	W	52	45.76	48.62
		A	43.22	64.52	47.65	A*	40.82	59.32	50.93
	p_E_		**0.003**	0.18	**0.001**		0.18	**0.021**	**0.007**
	p_C/E_		0.215	0.727	**0.022**		1	0.344	0.424
a*β*2GPI IgG	CLIA	Y	47.9	54.84	49.33	Y	48	54.24	51.38
		W	53.78	61.29	55.34	W	46	42.37	44.04
	p_C_		0.21	0.625	0.442		1	0.092	0.263
	ELISA	W*	33.61	74.19	42	W	26	67.8	48.63
		A	25.42	83.87	40.94	A*	22.45	79.66	53.71
	p_E_		**0.021**	0.25	**0.004**		0.687	**0.016**	**0.022**
	p_C/E_								
a*β*2GPI IgM	CLIA	#Y*	22.69	83.87	35.33	Y	20	77.97	51.37
		W	8.4	87.1	24.67	W*	10	91.53	54.13
	p_C_		**<0.001**	1	**<0.001**		0.125	**0.008**	**0.001**
	ELISA	W*	15.97	87.1	30.67	W	16	83.05	52.29
		A	8.47	93.55	26.17	A	6.12	93.22	53.71
	p_E_		**0.039**	0.625	**0.004**		**0.031**	0.219	**0.006**
	p_C/E_		0.077	1	**0.001**				
a*β*2GPI IgA	CLIA	Y	14.29	74.19	26.66	Y*	26	88.14	59.64
		W	13.45	83.87	28	W	20	88.14	56.88
	p_C_		1	0.375	0.344		0.453	1	**0.013**
	ELISA	W*	10.92	83.87	28.25	W	14	84.75	52.29
		A	6.78	93.55	24.83	A	6.12	89.83	51.85
	p_E_		0.125	0.375	**0.039**		0.289	0.25	0.065
	p_C/E_								
a*β*2GPI IgG or IgM or IgA	CLIA	Y	60.5	48.29	58	Y	58	44.01	50.46
		W	55.46	61.29	56.67	W	46	42.37	47.71
	p_C_		0.345	0.125	0.11		0.07	1	0.383
	ELISA	W	45.38	67.74	50	W	38	57.63	48.62
		A	34.75	77.42	57.63	A*	28.57	71.19	51.85
	p_E_		**0.008**	0.375	**0.003**		0.219	**0.002**	**0.002**
	p_C/E_								
a*β*2GPI IgG or IgM	CLIA	Y	60.5	51.61	58.67	Y	56	44.07	49.54
		W	55.46	61.29	56.67	W	46	42.37	44.04
	p_C_		0.345	0.375	0.163		0.18	1	0.523
	ELISA	W*	49.58	64.52	52.66	W	40	50.85	45.87
		A	40.68	74.19	47.65	A*	28.57	61.02	46.29
	p_E_		**0.021**	0.375	**0.007**		0.125	**0.031**	**0.003**
	p_C/E_								

P-values of sensitivity and specificity are calculated with McNemar test; significant results are marked bold.

P_C_, Comparison of CLIA results from different manufacturers. *Better results.

P_E_, Comparison of ELISA results from different manufacturers. *Better results.

P_C/E_, Comparison of better CLIA and ELISA results. #Best results.

Odds ratios (ORs) with 95% confidence intervals (CIs) are shown.

### Distribution of aPL Test Results

**Figure 1:**
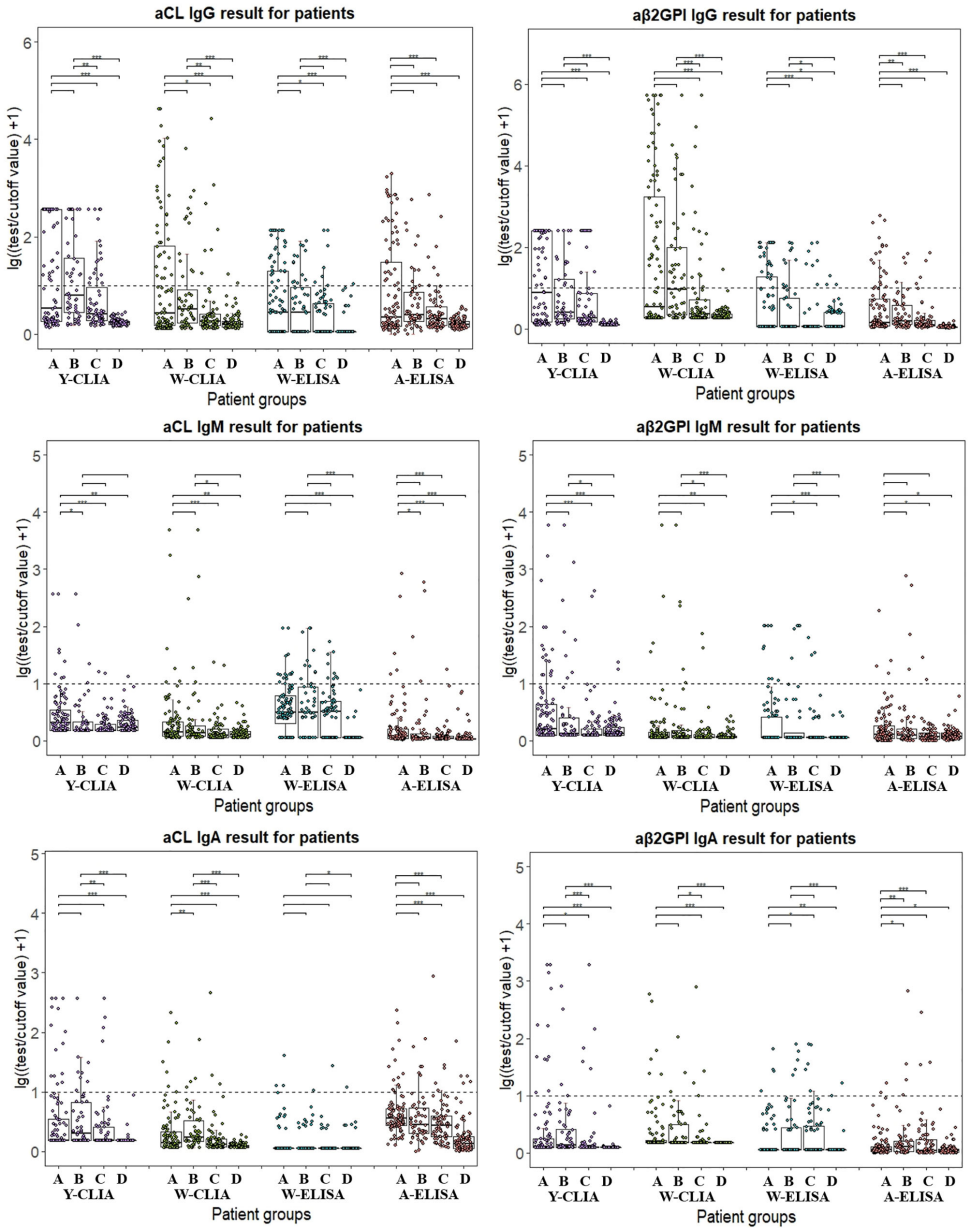
Distribution of aPL test results from different manufacturers among different patient groups. Test results are calculated in lg[(test result/cutoff value) +1]. A: PAPS, B: SAPS, C: SLE, D: Health control. Wilcox’s test is conducted comparing primary or secondary APS result to other patient groups. *P < 0.05, **P < 0.01, ***P < 0.001.

**Figure 2:**
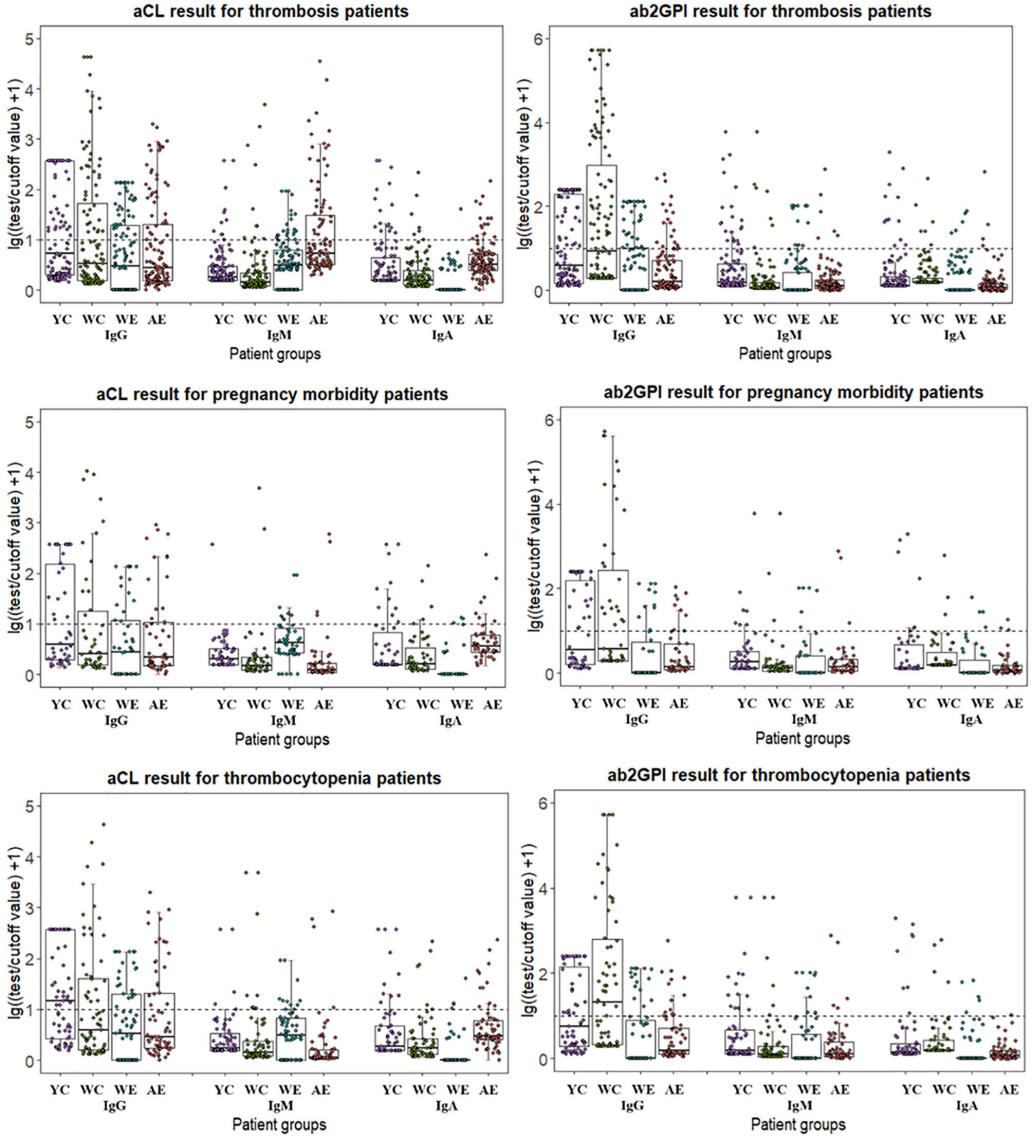
Distribution of aPL test results from different manufacturers for patients with different manifestations. Test results are calculated in lg[(test result/cutoff value) +1]. YC, Y-CLIA; WC, W-CLIA; WE, W-ELISA; AE, A-ELISA.

As different cut-off values were used by four test systems, the distribution of aPL test results from different manufacturers among patient groups were calculated with lg[(test result/cutoff value) +1] so that they could be visualized together as positive numbers in **Figure 1**. Patients positive for antibodies fell above the dotted line, and the range of distribution varied due to use of both test methods and limitation of test range for different antibodies. In general, W-CLIA had the widest range of test distribution, while W-ELISA had the narrowest. For Y-CLIA, test range limitation influenced distribution for three autoantibodies. The results of primary or secondary APS patients were compared to other groups and illustrated. Overall, most test systems could distinguish between APS patients and HC, while little significant difference was observed between PAPS and SAPS groups. For different antibodies, four test systems showed different strengths of differential diagnosis. For instance, W-CLIA was best at discrimination for aCL IgG, while A-ELISA was best at aCL IgM. Additionally, distribution of aPLs among clinical groups with the largest number of patients (*i.e.*, thrombosis, pregnancy morbidity, and thrombocytopenia) was also illustrated in **Figure 2**.

### Cross-Positivity Analysis for Four aPL in APS Patients

**Figure 3:**
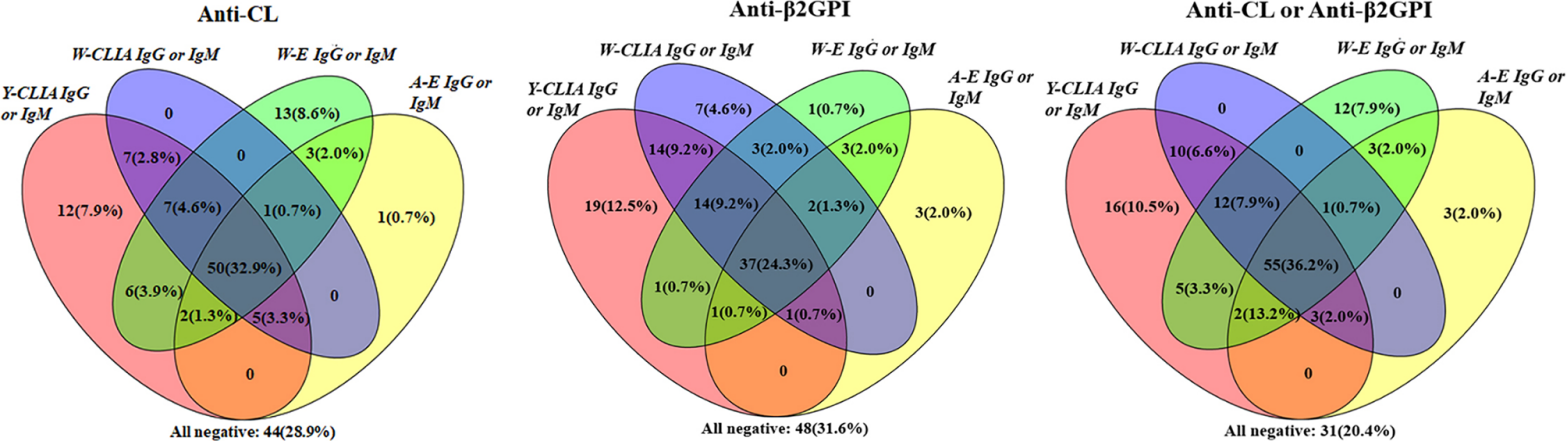
Cross-positivity among different tests for anti-CL, anti-*β*2GPI, and anti-CL or anti-*β*2GPI antibodies in APS patients (n = 152).

Among 152 patients, cross positivity for IgG or IgM of aCL and a*β*2GpI for each of the four test systems were demonstrated with Venn diagrams (**Figure 3**). For aCL, 50 (32.9%) patients were tested positive for IgG or IgM by all systems. There were 12 (7.9%) patients who were tested positive only by Y-CLIA, and 13 (8.6%) were tested positive only by W-ELISA. Similarly, for a*β*2GpI, 19 (12.5%) patients were test positive only by Y-CLIA, and seven (4.6%) were tested positive only by W-CLIA. When combining the positivity of aCL and a*β*2GpI, Y-CLIA identified the most amount of positive patients (totally 102, 67.8%), with the highest level of patients distinguished only by the system (16, 10.5%).

### Clinical Manifestations Prediction for the Test Systems

The correlation of different aPL levels by all four test systems with non-criteria clinical manifestations was further explored, with significant results presented in **Table 5**. Thrombocytopenia was associated with the greatest number of antibody positivity (aCL IgG by Y-CLIA, aCL IgM/a*β*2GpI IgG/a*β*2GpI IgM by W-CLIA, and a*β*2GpI IgM by W-ELISA). Significant association was also observed for APSN, PVT, PE, DVT, and positivity of some autoantibodies by certain test systems. Little association was observed between IgA with any clinical features.

**Table 5:** Significant association between non-criteria clinical manifestations and aPL levels by different test systems.

	Significant association	χ^2^	*p
YCLIA	TP & aCL IgG	6.935	0.008
	APSN & a*β*2GPI IgG	5.644	0.026
	PVT & aCL IgG	5.308	0.027
WCLIA	PE & aCL IgG	7.636	0.006
	TP & aCL IgM	5.043	0.025
	TP & a*β*2GPI IgG	4.511	0.034
	TP & a*β*2GPI IgM	4.679	0.031
WELISA	TP & a*β*2GPI IgM	4.913	0.027
	APSN & a*β*2GPI IgG	7.369	0.013
	APSN & aCL IgG	9.007	0.005
AELISA	DVT & a*β*2GPI IgM	5.466	0.035

*Fisher exact tests were conducted if needed. TP, thrombocytopenia; APSN, antiphospholipid syndrome nephropathy; PVT, portal vein thrombosis; PE, pulmonary emphysema; DVT, deep vein thrombosis.

## Discussion

APS is an autoimmune disease featuring thrombosis and/or pregnancy morbidity which may lead to severe consequences. In order to accurately identify APS patients and provide timely clinical intervention, a detection system with high sensitivity and specificity is required. In this study, the diagnostic and analytic performances of four commercial assays were compared in detecting IgG/IgM/IgA for aCL and a*β*2GPI antibodies. In brief, CLIA by YHLO Biotech Co. was considered as the system with the best predictive power, where 58.55 and 57.89% of APS patients were positive for aCL or a*β*2GPI for at least one antibodies (IgG or IgM or IgA). Y-CLIA also identified the greatest number of patients (67.8%) positive for aCL or a*β*2GpI IgG or IgM, with the highest level of patients distinguished only by the system (16, 10.5%). Nevertheless, for Y-CLIA, little correlation of antibodies’ positivity result with thrombosis or pregnancy complication was observed. In addition, the greatest number of double/triple patients was detected by Y-CLIA. Concerning clinical manifestations, a significant association was observed between W-CLIA and TP/PE, Y-CLIA and TP, as well as combined results with TP/PE/thrombosis. Overall, CLIA showed better performance characteristics than traditional ELISA test systems.

Many previous studies have found poor agreement among different aPL assay platforms (5, 18), which may result from various factors. As shown in **Table 2**, depending on the coating method for solid phase, antibodies detected would either bind to cardiolipin or bind directly to *β*2GPI. In addition, different conjugates were applied for signal detection. A lack of universal internal standards for calibration further increased the chance of discrepancy. In addition, different cut-off values were chosen, as they stem from heterogenous reference sample groups in the original calculation. Thus, it might be better to choose the same appropriate reference population among all platforms and utilize an in-house 99th percentile cut-off value, which had been recommended by all manufacturers and confirmed by previous studies (19, 20). Nevertheless, due to the restriction of subjects, this study still chose the cut-off values provided by platform instructions respectively, which might not reflect the distribution characteristics of the disease population. Compared to ELISA, automated CLIA has the advantage of increasing reproducibility, reducing hands-on time as well as avoiding manual error, which had been proved by some previous studies.

With regard to the predictive value of aPLs detected by the four systems, Y-CLIA stood out as the best. **Table 3** indicated that the sensitivity, specificity, accuracy, and Youden index were higher for Y-CLIA among each comparison whenever a significant difference was found. As for ELISAs, W-ELISA had higher predictive power for most aPLs compared to A-ELISA. However, no single detection system had stably shown better performance for all aPLs. Distribution of aPL test results in **Figure 1** further reflected this inconsistency. Y-CLIA did not show better ability at distinguishing PAPS or SPAS from SLE or HC groups compared to other systems. Indeed, it had been estimated in previous studies that around 40% of patients with SLE have aPL, and APS may develop in up to 50–70% of patients with both SLE and aPLs (21). Thus, although Y-CLIA could be recommended for APS diagnosis, other systems may provide additive value for each individual aPL in differentiation, especially when SLE was involved. The predictive power of criterial manifestations indicated that besides serology diagnosis, different systems had respective strengths in predicting associate events. W-CLIA was more sensitive and accurate for thrombosis, while results from A-ELISA were more specific and accurate for pregnancy-related outcomes. Since APS diagnosis relied both on clinical and experimental criteria, inclusion of more test systems was still of great importance.

As IgG or IgM of aCL and a*β*2GpI was part of the standard diagnostic criteria, cross-positivity analysis was conducted, which revealed that Y-CLIA identified the most number of patients test positive overall. However, other systems were still of great value for different aPLs, as 8.6% of aCL and 4.6% of a*β*2GpI were tested positive only by W-ELISA or W-CLIA. which suggested that a combination of more test systems could increase the sensitivity of APS diagnosis. In the clinic, patients may remain persistently negative for criteria aPLs yet show typical APS clinical manifestations (defined as seronegative APS, SNAPS) (22). Alternate testing platforms could assist in final diagnosis for SNAPS patients.

According to the European League Against Rheumatism (EULAR) guidelines for APS, high-risk profiles for APS is defined as a positive LA test, the presence of double (any combination of LA, aCL or a*β*2GPI antibodies) or triple (all three subtypes) aPL positivity, or the presence of persistently high aPL titers (23). It is crucial to recognize these high-risk patients in order for the early prevention of thrombotic and obstetric events (24). Thus, a cross-positivity analysis was conducted to evaluate the ability of four test systems in identifying high-risk patients concerning aCL/a*β*2GPI detection (result not shown). For double-positive patients, among 94 patients (61.84%) positive for LA and aCL, eleven and nine were detected positive only by Y-CLIA and W-ELISA respectively. Among 92 patients (60.53%) positive for LA and a*β*2GPI, seven and six were detected positive only by Y-CLIA and W-CLIA respectively. For 77 triple-positive patients (50.66%), nine were detected positive only by Y-CLIA and two by W-CLIA. The result suggested that a combination of more test systems could increase the sensitivity of high-risk identification for APS.

Finally, the results of different aPL isotypes tested by four systems were explored of their association with non-criteria clinical manifestations. Thrombocytopenia was associated with the greatest number of antibody positivity, and significant results were also observed for APSN, PVT, PE, and DVT. However, no other significant association was observed for other clinical features or IgA isotype. Similar results could be observed in a study conducted by us recently in a large cohort with more than 7,000 patients (25). It had been reported that the prevalence of thrombocytopenia was 20 to 46% as a manifestation of primary APS, probably because aCL may bind activated platelet membranes and cause platelet destruction (26). Although the correlation between aPLs and thrombosis or pregnancy events has been confirmed by a number of studies (27–29), conflicting results have also been observed in other reports (9, 30). In our study, venous thrombosis events (PVT, PE, and DVT) showed more correlation with aPL positivity, while little significant relationship was found with poor pregnancy outcomes. It should be noted that the number of patients with most of the recorded clinical manifestations was small (**Figure 1**). Consequently, the results might be strongly influenced by patient heterogeneity including age, gender, or other factors.

All in all, this study confirmed the advantage of using CLIA testing systems for aPL detection, with higher predictive power and better ability at identifying both low-titer suspected and multi-positive high-risk patients. In the future, with the reduction of test apparatus cost, fully automated CLIA could replace ELISA in the laboratory testing of aPLs for APS diagnosis and monitor. For the local population in China, Y-CLIA would be a more suitable choice concerning commercially available testing systems. Our study has some limitations. Recommended cut-off values were used and not calculated with the local population, which might decrease precision in sequential analysis. Correlation between autoantibodies and clinical manifestations, especially obstetrical related events, still needs examination. Larger sample size and inclusion of patients with a wider range of associated diseases or clinical features, as well as more high-risk patients (double/triple-positive), could further complement the study. The predictive performance of the selected test system (Y-CLIA) also needs further confirmation.

## Conclusion

In conclusion, CLIA was considered a better platform for IgG/IgM/IgA aCL and a*β*2GPI detection in APS diagnosis. Additionally, a combination of other detection platforms could assist in clinical diagnosis and differential diagnosis, increase the ability to exclude SNAPS, as well as identify high-risk patients.

## Data Availability Statement

The raw data supporting the conclusions of this article will be made available by the authors, without undue reservation.

## Ethics Statement

The studies involving human participants were reviewed and approved by the Medical Ethics Committee of Peking Union Medical College Hospital. The patients/participants provided their written informed consent to participate in this study.

## Author Contributions

All authors were involved in the design of this study. CH, SL, ZX, HY, HJ, and JZ contributed to the collection of blood samples and other experimental procedures. YS and WQ were involved in data collection and pre-processing. CH and SL analyzed the data and wrote the manuscript. JZ, QW, XT, ML, and YZ contributed to the recruitment of patients and evaluation of clinical data. All authors contributed to the article and approved the submitted version.

## Funding

This study was supported by the National Key Research and Development Program of China (2019YFC0840603, 2017YFC0907601, and 2017YFC0907602), the National Natural Science Foundation of China (81771780), and the CAMS Initiative for Innovative Medicine (2017-I2M-3-001 and 2019-I2M-2-008).

## Conflict of Interest

The authors declare that the research was conducted in the absence of any commercial or financial relationships that could be construed as a potential conflict of interest.
